# Spatial separation of two different pathways accounting for the generation of calcium signals in astrocytes

**DOI:** 10.1371/journal.pcbi.1005377

**Published:** 2017-02-13

**Authors:** Franziska Oschmann, Konstantin Mergenthaler, Evelyn Jungnickel, Klaus Obermayer

**Affiliations:** 1 Technische Universität Berlin, Neural Information Processing Group, Berlin, Germany; 2 Bernstein Center for Computational Neuroscience, Berlin, Germany; University of Virginia, UNITED STATES

## Abstract

Astrocytes integrate and process synaptic information and exhibit calcium (Ca^2+^) signals in response to incoming information from neighboring synapses. The generation of Ca^2+^ signals is mostly attributed to Ca^2+^ release from internal Ca^2+^ stores evoked by an elevated metabotropic glutamate receptor (mGluR) activity. Different experimental results associated the generation of Ca^2+^ signals to the activity of the glutamate transporter (GluT). The GluT itself does not influence the intracellular Ca^2+^ concentration, but it indirectly activates Ca^2+^ entry over the membrane. A closer look into Ca^2+^ signaling in different astrocytic compartments revealed a spatial separation of those two pathways. Ca^2+^ signals in the soma are mainly generated by Ca^2+^ release from internal Ca^2+^ stores (mGluR-dependent pathway). In astrocytic compartments close to the synapse most Ca^2+^ signals are evoked by Ca^2+^ entry over the plasma membrane (GluT-dependent pathway). This assumption is supported by the finding, that the volume ratio between the internal Ca^2+^ store and the intracellular space decreases from the soma towards the synapse. We extended a model for mGluR-dependent Ca^2+^ signals in astrocytes with the GluT-dependent pathway. Additionally, we included the volume ratio between the internal Ca^2+^ store and the intracellular compartment into the model in order to analyze Ca^2+^ signals either in the soma or close to the synapse. Our model results confirm the spatial separation of the mGluR- and GluT-dependent pathways along the astrocytic process. The model allows to study the binary Ca^2+^ response during a block of either of both pathways. Moreover, the model contributes to a better understanding of the impact of channel densities on the interaction of both pathways and on the Ca^2+^ signal.

## Author summary

Astrocytes are considered as active partners in neural information processing, because they integrate and process synaptic information and control synaptic transmission. Neuronal transmitter release induces the generation of Ca^2+^ signals in astrocytes. The functional role of astrocytic Ca^2+^ signals is still under debate. However, experimental results were able to show that astrocytic Ca^2+^ signaling acts to control local network activity, which plays an important role in diseases like epilepsy. Thus, it is of special interest to investigate the underlying mechanisms for Ca^2+^ signals in astrocytes in order to understand the role of astrocytes in neural network activity. Two different mechanisms are known to be responsible for the generation of Ca^2+^ signals in astrocytes. These mechanisms are the release of Ca^2+^ from internal Ca^2+^ stores and the entry of Ca^2+^ through the plasma membrane. We studied the interaction of those two different mechanisms for the generation of Ca^2+^ signals and found that these mechanisms are spatially separated along the astrocytic processes.

## Introduction

**Fig 1:**
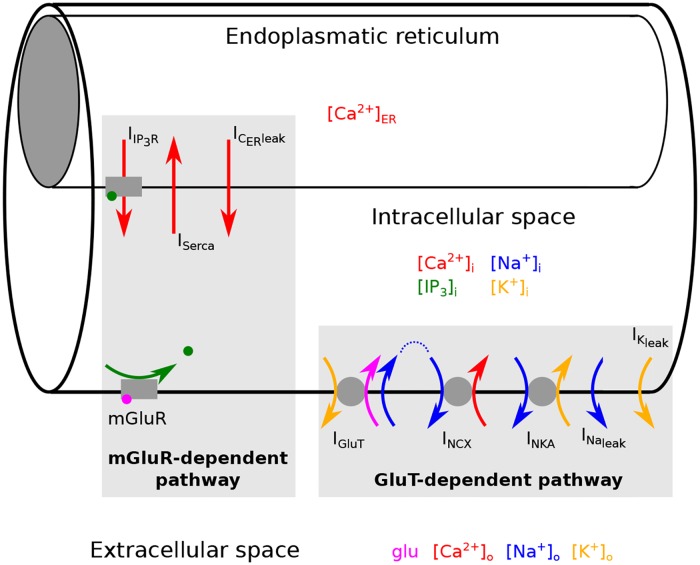
Generation of Ca^2+^ signals in an astrocyte. We consider astrocytic compartments which consist of three parts: the intracellular space, the internal Ca^2+^ store (endoplasmatic reticulum) and the extracellular space. Ca^2+^ signals in the intracellular space are generated by two different pathways: the metabotropic glutamate receptor (mGluR)-dependent pathway and the glutamate transporter (GluT)-dependent pathway. The mGluR-dependent pathway describes the glutamate dependent production of IP_3_, which then evokes IP_3_ and Ca^2+^ dependent exchange of Ca^2+^ between the intracellular space and the endoplasmatic reticulum. The GluT-dependent pathway describes the GluT driven transport of Ca^2+^ between the extracellular and the intracellular space.

Astrocytes integrate and process synaptic information and by doing so generate calcium (Ca^2+^) signals in response to neurotransmitter release from neighboring synapses [1]. Ca^2+^ signals in astrocytes are largely attributed to an elevated metabotropic glutamate receptor (mGluR) activity, which stimulates the phospholipase C and the production of the second messenger inositol trisphosphate (IP_3_). The binding of IP_3_ to receptors at internal Ca^2+^ stores (endoplasmatic reticulum) induces IP_3_ and Ca^2+^-dependent Ca^2+^ release into the intracellular space [2–7] (see mGluR-dependent pathway in Fig 1).

Experimental results, however, showed not only a clear attenuation of the Ca^2+^ signal during an inhibition of the mGluR, but also during a block of the glutamate transporter (GluT) [7, 8]. The glutamate transporter itself does not influence the intracellular Ca^2+^ concentration, but it indirectly activates Ca^2+^ entry over the membrane mediated by the Na^+^/Ca^2+^ exchanger [9] (see GluT-dependent pathway in Fig 1). The uptake of one glutamate molecule mediated by the glutamate transporter is accompanied by the transport of three sodium (Na^+^) ions into the astrocyte and one potassium (K^+^) ion out of the astrocyte. An inwardly directed Na^+^ gradient and an outwardly directed K^+^ gradient promote the glutamate uptake by the glutamate transporter and glutamate accumulation in the astrocyte. The Na^+^-K^+^-ATPase maintains the Na^+^-K^+^ concentration gradient and favors the glutamate transport [10]. In close proximity to glutamate transporters high concentrations of Na^+^/Ca^2+^ exchangers have been observed [9]. During a rapid rise of the Na^+^ concentration the Na^+^/Ca^2+^ exchanger works in the reverse mode and transports Na^+^ out of the astrocyte while transporting Ca^2+^ into the astrocyte. Thereby the Na^+^/Ca^2+^ exchanger serves as an additional transient source of Ca^2+^ and the intracellular Ca^2+^ concentration increases [9].

**Fig 2:**
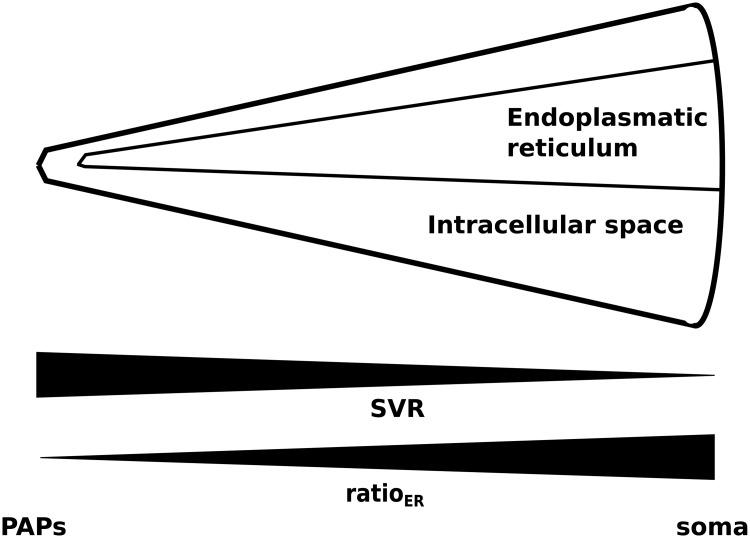
Changes of the astrocytic surface to volume ratio (SVR) and the volume ratio of internal Ca^2+^ stores compared to the intracellular space (ratio_ER_) for astrocytic compartments along the astrocytic process. A small ratio_ER_ corresponds to astrocytic compartments close to the synapse (perisynaptic astrocytic processes (PAPs)) and a high ratio_ER_ corresponds to astrocytic regions at the soma.

Therefore, at least two different mechanisms contribute to the generation of Ca^2+^ signals in astrocytes. A closer look into Ca^2+^ signaling in different astrocytic compartments revealed a spatial separation of those two pathways. In the soma Ca^2+^ signals are mainly evoked on the mGluR-dependent pathway, whereas in perisynaptic astrocytic processes (PAPs) most Ca^2+^ signals are evoked by Ca^2+^ entry over the plasma membrane [11]. These results are supported by the finding, that astrocytic compartments close to the synapse are devoid of internal Ca^2+^ stores and the volume ratio of internal Ca^2+^ stores compared to the intracellular space increases towards the soma. Moreover, the surface volume ratio decreases along the astrocytic process from the PAPs towards the soma, because processes become increasingly thinner (see Fig 2) [12].

Based on the findings cited above we hypothesized that the underlying mechanisms for Ca^2+^ signals differ between astrocytic compartments. The mGluR-dependent pathway is mainly present close to the astrocytic soma, while the GluT-dependent pathway dominates Ca^2+^ signals in PAPs. So far most mathematical models attribute astrocytic Ca^2+^ dynamics solely to mGluRs and neglect Ca^2+^ entry through the membrane. In order to test whether the Na^+^/Ca^2+^ exchanger serves as a source for Ca^2+^ signals in PAPs, we propose a mathematical model, which incorporates glutamate driven Ca^2+^ responses evoked by simultaneous binding of glutamate to mGluR’s and transport of glutamate by GluT while taking the volume ratio of internal Ca^2+^ stores into account. With the help of the model we investigated how the volume ratio between the internal Ca^2+^ store and the intracellular space affects Ca^2+^ signaling evoked on the mGluR- and GluT-dependent pathway in different astrocytic compartments along astrocytic processes from the synapse towards the soma.

## Methods

We used a system of ordinary differential equations to describe the changes of the ion concentrations, the membrane voltage and the concentration of IP_3_ in a single astrocytic compartment (see Fig 1) of an astrocytic process. Glutamate dependent Ca^2+^ signals are evoked through two different pathways (see Fig 1). One pathway is driven by the activity of the metabotropic glutamate receptor (mGluR-dependent pathway). The other depends on the activity of the glutamate transporter (GluT-dependent pathway). In the mGluR-dependent pathway glutamate binds to the metabotropic glutamate receptors (mGluR) leading to an enhanced production of the second messenger IP_3_ and the subsequent IP_3_ dependent Ca^2+^ release from the internal Ca^2+^ store (endoplasmatic reticulum). The exchange of Ca^2+^ between the endoplasmatic reticulum (ER) and the intracellular space is mediated by three currents: the IP_3_ receptor current (I_IP_3_R_), which describes the IP_3_ dependent Ca^2+^ release from the ER, the Ca^2+^ current of the SERCA pump (I_Serca_), which transports Ca^2+^ back into the ER, and a Ca^2+^ leak current (I_C_ER__leak__). The IP_3_ receptor channel current is influenced by the concentration of the second messenger IP_3_, by the fraction h of active IP_3_ receptor channels, and by the Ca^2+^ concentration itself. The GluT-dependent pathway describes the transport of Ca^2+^ through the membrane driven by the activity of the glutamate transporter (GluT). This pathway includes the glutamate transporter, the Na^+^/K^+^-ATPase (NKA), the Na^+^/Ca^2+^ exchanger (NCX), and the Na^+^ and K^+^ leak currents. The Ca^2+^ transport through the membrane is influenced by the intra- and extracellular Ca^2+^, Na^+^ and K^+^ concentrations, and the membrane voltage V.

### Geometry of the astrocytic model compartment

We consider small astrocytic compartments, which have a cylindrical shape. Each astrocytic compartment consists of three parts: the internal Ca^2+^ store (endoplasmatic reticulum), the intracellular space, and the extracellular space (see Fig 1). The internal Ca^2+^ store and the intracellular space are considered as two cylinders with different diameter, which lie within each other. The volume of the intracellular space includes the volume of the internal Ca^2+^ store. The intracellular space is surrounded by the extracellular space. The volume of the extracellular space is set equal to the volume of the intracellular space. Flow of ions to neighboring compartments is not considered. Thus, only the curved surface area of the cylinder is considered.

For the change of the ion concentration within the intracellular space or the internal Ca^2+^ store (see Eq 2), we consider the sum of all ionic currents carrying the respective ion (∑*I*_*ion*_) multiplied with the area *A*, the ionic current is flowing through, and divided by the volume *Vol* of the space the ions are located in. Both *A* and *Vol* are scaled by the length *l* of the compartment. Therefore, the fraction $\frac{A}{Vol}$ does not depend on *l* and lateral diffusion of ions was neglected.

For each astrocytic compartment the surface area and the volume of both the internal Ca^2+^ store and the intracellular space change along the astrocytic process. The diameter of the intracellular space increases from astrocytic compartments close to the synapse towards astrocytic compartments at the soma (see Fig 2). Thus, the surface area and the volume of the intracellular space increase from the synapse to the soma, but the surface volume ratio (SVR) decreases. The volume ratio between the internal Ca^2+^ store and the intracellular space increases from astrocytic compartments close to the synapse towards astrocytic compartments at the soma. Astrocytic compartments close to the synapse do not contain internal Ca^2+^ stores (ratio_ER_ = 0) (see Fig 2).

Within a single astrocytic compartment the diameter of the internal Ca^2+^ store is smaller than the diameter of the intracellular space. The volume of the internal Ca^2+^ store is equal to the volume of the intracellular space reduced by the factor ratio_ER_. Consequently, the surface area of the internal Ca^2+^ store is reduced by the factor $\sqrt{ratio_{ER}}$ compared to the surface area of the intracellular space. Thus, the volume ratio between the internal Ca^2+^ store and the intracellular space determines the change of the surface volume ratio ($SVR=\frac{A}{Vol}$) of the internal Ca^2+^ store along the astrocytic process.

**Fig 3:**
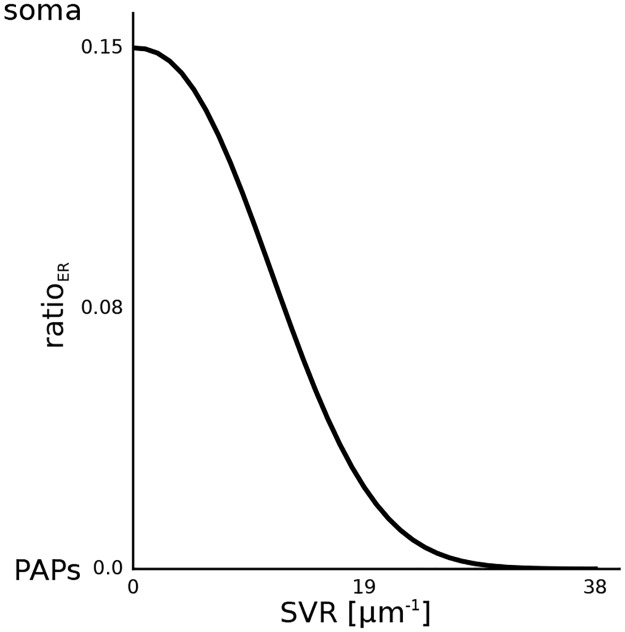
The volume ratio of the endoplasmatic reticulum (ER) as a function of the surface volume ratio (SVR). The data has been adapted from [12].

Along the astrocyte process, the surface volume ratio (SVR) and the volume ratio between the internal Ca^2+^ store and the intracellular space depend on each other, and the relationship (see [12] and Fig 3) is quantified by:

$$\begin{array}{c} ratio_{ER}=0.15\cdot e^{-{\left(0.002\mu m\cdot SVR\right)}^{2.32}} \end{array}$$ (1)

### Dynamics of the ion concentrations, the membrane voltage and the concentration of IP_3_

#### Dynamics of ion concentrations

The change of the ion concentration is given by:

$$\begin{array}{c} \frac{d[ion]}{dt}=\frac{A}{F\cdot Vol}\cdot \sum I_{ion} \end{array}$$ (2)

and depends on the sum of all ionic currents carrying the respective ion (∑*I*_*ion*_) multiplied with the area (*A*), the ionic currents are flowing through, and divided by the volume (*Vol*) of the space the ions are located in and the Faraday constant (F). The change of the intracellular Ca^2+^ concentration is determined by currents crossing either the membrane of the internal Ca^2+^ store or of the outer cell membrane. For that reason the change of the intracellular Ca^2+^ concentration reads as follows:

$$\begin{array}{c} \frac{d{\left[Ca^{2+}\right]}_{i}}{dt}=\frac{A}{F\cdot Vol}\cdot I_{NCX}+\frac{A\cdot \sqrt{ratio_{ER}}}{F\cdot Vol}\cdot \left(I_{IP_{3}R}-I_{Serca}+I_{C_{ER}leak}\right), \end{array}$$ (3)

where *A* denotes the area of the outer cell membrane, $A\cdot \sqrt{ratio_{ER}}$ is the area of the internal Ca^2+^ store and the volume of the intracellular space is defined as *Vol*.

The change of the Ca^2+^ concentration in the ER is determined by currents crossing the membrane of the ER:

$$\begin{array}{cc} \frac{d{\left[Ca^{2+}\right]}_{ER}}{dt} & =\frac{A\cdot \sqrt{ratio_{ER}}}{F\cdot Vol\cdot ratio_{ER}}\cdot \left(-I_{IP_{3}R}+I_{Serca}-I_{C_{ER}leak}\right), \end{array}$$

here $A\cdot \sqrt{ratio_{ER}}$ and *Vol* ⋅ *ratio*_*ER*_ describe the area and the volume of the internal Ca^2+^ store, respectively.

The change of the intracellular Na^+^ and K^+^ concentrations are described by the following equations:

$$\begin{array}{cc} \frac{d{\left[Na^{+}\right]}_{i}}{dt} & =\frac{A}{F\cdot Vol}\cdot \left(3I_{GluT}-3I_{NKA}-3I_{NCX}-I_{Na_{leak}}\right)\\ \frac{d{\left[K^{+}\right]}_{i}}{dt} & =\frac{A}{F\cdot Vol}\cdot \left(-I_{GluT}+2I_{NKA}-I_{K_{leak}}\right). \end{array}$$

#### Dynamics of the membrane voltage

The change of the membrane voltage V is determined by:

$$\begin{array}{ll} \frac{dV}{dt} & =-\frac{1}{C_{m}}(-2I_{IP_{3}R}+2I_{Serca}-2I_{C_{ER}leak}+I_{NCX}- \end{array}2I_{GluT}+I_{NKA}+I_{Na_{leak}}+I_{K_{leak}}).$$

The right hand side of the equation consists of the sum of all ionic membrane currents with the consideration of carried charges per ion (see Fig 1). C_m_ is the membrane capacitance. Note, that the transport of sodium and potassium mediated by the glutamate transporter lead to a net transfer of two positive charges per cycle across the membrane.

#### Extracellular ion concentrations

The changes of the extracellular Ca^2+^, Na^+^ and K^+^ concentrations are determined by:

$$\begin{array}{c} {\left[Ca^{2+}\right]}_{o}-{\left[Ca^{2+}\right]}_{o_{rest}}={\left[Ca^{2+}\right]}_{i}-{\left[Ca^{2+}\right]}_{i_{rest}}+{\left[Ca^{2+}\right]}_{ER_{rest}} \end{array}-{\left[Ca^{2+}\right]}_{ER}$$ (4)

$$\begin{array}{c} {\left[Na^{+}\right]}_{o}-{{\left[Na^{+}\right]}_{o}}_{rest}={{\left[Na^{+}\right]}_{i}}_{rest}-{\left[Na^{+}\right]}_{i} \end{array}$$ (5)

$$\begin{array}{c} {\left[K^{+}\right]}_{o}-{[K^{+}{]}_{o}}_{rest}={{\left[K^{+}\right]}_{i}}_{rest}-{\left[K^{+}\right]}_{i}. \end{array}$$ (6)

We calculated the extracellular concentration as a function of the intracellular concentration under the assumption that the volume of the intracellular and extracellular space of an astrocytic compartment are the same and the overall concentration in the intracellular and the extracellular space of an astrocytic compartment stays constant. Values of model parameters can be found in Table 1.

**Table 1:** Initial values of the ion concentrations, the membrane voltage, IP_3_ and the fraction of the activated IP_3_ receptor channels. For the calculation of [Ca^2+^]_ER_, [IP_3_]_i_ and h see Model section **Model parameter values**.

Parameter	Value	Source
[*Ca*^2+^]_*i*_	0.073 *μ*M	[23]
[*Ca*^2+^]_*ER*_	25 *μ*M	see text
[*Ca*^2+^]_*o*_	1800 *μ*M	[18]
[*Na*^+^]_*i*_	15 mM	[19]
[*Na*^+^]_*o*_	145 mM	[19]
[*K*^+^]_*i*_	100 mM	[19]
[*K*^+^]_*o*_	3 mM	[19]
V	-85 mV	[24]
[*IP*_3_]_*i*_	0.15659 *μ*M	see text
h	0.7892	see text

#### IP_3_ production and degradation

The concentration change of the second messenger IP_3_ is determined by the production and degradation of IP_3_. The production is mediated by the phosphoinositide-specific phospholipase C *β* (PLC*β*) and the phosphoinositide-specific phospholipase C *δ* (PLC*δ*). The degradation is mediated by the IP_3_ 3-kinase (IP_3_-3K) and the inositol polyphosphate 5-phosphatase (IP-5P) [13].

$$\begin{array}{ll} \frac{d{\left[IP_{3}\right]}_{i}}{dt} & =prod_{PLC\beta }+prod_{PLC\delta }-degr_{IP_{3}-3K}-degr_{IP-5P}\\ prod_{PLC\beta } & =v_{\beta }\cdot \frac{g^{0.7}}{g^{0.7}+{\left(K_{R}+K_{p}\cdot \frac{{\left[Ca^{2+}\right]}_{i}}{{\left[Ca^{2+}\right]}_{i}+K_{\pi }}\right)}^{0.7}}\\ prod_{PLC\delta } & =\frac{v_{\delta }}{1+\frac{{\left[IP_{3}\right]}_{i}}{\kappa_{\delta }}}\cdot \frac{{\left[Ca^{2+}\right]}_{i}^{2}}{{\left[Ca^{2+}\right]}_{i}^{2}+K_{PLC\delta }^{2}}\\ degr_{IP_{3}-3K} & =v_{3K}\cdot \frac{{\left[Ca^{2+}\right]}_{i}^{4}}{{\left[Ca^{2+}\right]}_{i}^{4}+K_{D}^{4}}\cdot \frac{{\left[IP_{3}\right]}_{i}}{{\left[IP_{3}\right]}_{i}+K_{3}}\\ degr_{IP-5P} & =r_{5p}\cdot {\left[IP_{3}\right]}_{i}. \end{array}$$

The production of IP_3_ by the phosphoinositide-specific phospholipase C (PLC) *β* is linked to the level of the extracellular glutamate concentration g. The maximal rate of IP_3_ production by PLC*β* is described by v_*β*_ and the glutamate affinity of the receptor is set by K_R_. K_p_ is the Ca^2+^/PLC-dependent inhibition factor and K_*π*_ determines the Ca^2+^ affinity of PLC.

The maximal rate of IP_3_ production by PLC*δ* is described by v_*δ*_. The activity of PLC*δ* is inhibited according to the inhibition constant k_*δ*_. The Ca^2+^ affinity of PLC*δ* is set by K_PLC*δ*_.

The maximal degradation rate of IP_3_ by IP_3_-3K is determined by v_3K_. K_D_ is the Ca^2+^ affinity of IP_3_-3K and K_3_ is the IP_3_ affinity of IP_3_-3K.

The degradation of IP_3_ through dephosphorylation by the inositol polyphosphate 5-phosphatase (IP-5P) depends on the maximal rate, r_5P_, of degradation by IP-5P. Values of model parameters can be found in Table 2.

**Table 2:** Model parameters for the production and degradation of IP_3_. IP_3_ production is mediated by PLC*β* and PLC*δ* and IP_3_ degradation is mediated by IP_3_—3K and IP—5P.

Parameter	Value	Source
**IP_3_ production by PLC*β***		
v_*β*_	0.05 $\frac{\mu M}{s}$	[13]
K_R_	1.3 *μ*M	[13]
K_p_	10 *μ*M	[13]
K_*π*_	0.6 *μ*M	[13]
**IP_3_ production by PLC*δ***		
v_*δ*_	0.02 $\frac{\mu M}{s}$	[13]
k_*δ*_	1.5 *μ*M	[13]
K_PLC*δ*_	0.1 *μ*M	[13]
**IP_3_ degradation by IP_3_—3K**		
v_3K_	2 $\frac{\mu M}{s}$	[13]
K_D_	0.7 *μ*M	[13]
K_3_	1 *μ*M	[13]
**IP_3_ degradation by IP—5P**		
r_5P_	0.04 $\frac{1}{s}$	[13]

### Currents

#### Intracellular dynamics

*Ca^2+^ current through IP_3_ receptor channels*. The Ca^2+^ current through the IP_3_ receptor channel was taken from [14]:

$$\begin{array}{c} \begin{array}{cc} I_{IP_{3}R} & =\frac{F\cdot Vol}{A}\cdot r_{C}\cdot {\left(\frac{{\left[IP_{3}\right]}_{i}}{{\left[IP_{3}\right]}_{i}+d_{1}}\right)}^{3}\cdot {\left(\frac{{\left[Ca^{2+}\right]}_{i}}{{\left[Ca^{2+}\right]}_{i}+d_{5}}\right)}^{3}\cdot h^{3}\cdot \left({\left[Ca^{2+}\right]}_{ER}-{\left[Ca^{2+}\right]}_{i}\right). \end{array} \end{array}$$ (7)

r_C_ determines the maximal rate of transported Ca^2+^ ions. The dissociation of IP_3_ and Ca^2+^ by the channels’ subunits is determined by d_1_ and d_5_.

The probability of the channel to be in the open state is characterized by the term ${\left(\frac{{\left[IP_{3}\right]}_{i}}{{\left[IP_{3}\right]}_{i}+d_{1}}\right)}^{3}\cdot {\left(\frac{{\left[Ca^{2+}\right]}_{i}}{{\left[Ca^{2+}\right]}_{i}+d_{5}}\right)}^{3}\cdot h^{3}$ and depends on the intracellular IP_3_ concentration, the intracellular Ca^2+^ concentration and the fraction h of activated IP_3_ receptor channels. The channel can either be in the activated or the inactivated state. As proposed in [14] the channel is in the activated state when one Ca^2+^ ion and one IP_3_ molecule bind to two out of the three subunits of the channel. The channel is in the inactivated state when a second Ca^2+^ ion binds to the third subunit. The current strength is proportional to the Ca^2+^ gradient between the ER and the intracellular space, ([Ca^2+^]_ER_—[Ca^2+^]_i_). In order to relate the current strength to the volume of the intracellular space, the current is multiplied with the volume *Vol*. The current is normalized by the area *A*. Values of model parameters can be found in Table 3.

**Table 3:** Model parameters for the Ca^2+^ currents through the membrane of the endoplasmatic reticulum: The IP_3_ receptor channel, the SERCA pump and the Ca^2+^ leak current.

Parameter	Value	Source
**IP_3_ receptor channel**		
r_C_	6 $\frac{1}{s}$	[13]
d_1_	0.13 *μ*M	[13]
d_5_	0.08234 *μ*M	[13]
**SERCA pump**		
v_ER_	4 $\frac{\mu M}{s}$	[25, 26]
K_ER_	0.1 *μ*M	[13]
**Ca^2+^ leak**		
r_L_	0.11 $\frac{1}{s}$	[13]

*Activation of IP_3_ receptor channels*. The fraction h of activated IP_3_ receptor channels was taken from [14],

$$\begin{array}{c} \frac{dh}{dt}=a_{2}\cdot \left(d_{2}\cdot \frac{{\left[IP_{3}\right]}_{i}+d_{1}}{{\left[IP_{3}\right]}_{i}+d_{3}}\cdot \left(1-h\right)-h\cdot {\left[Ca^{2+}\right]}_{i}\right) \end{array}$$ (8)

a_2_ determines the IP_3_R binding rate for Ca^2+^ inhibition. The inactivation dissociation constants of Ca^2+^ and IP_3_ are d_2_ and d_3_, respectively. Values of model parameters can be found in Table 4.

**Table 4:** Model parameters for the dynamics of the fraction h of activated IP_3_ receptor channels.

Parameter	Value	Source
a_2_	0.2 $\frac{1}{s}$	[13]
d_2_	1.049 *μ*M	[13]
d_3_	0.9434 *μ*M	[13]

*SERCA pump*. The transport of Ca^2+^ ions into the endoplasmatic reticulum mediated by the SERCA pump was taken from [14],

$$\begin{array}{c} I_{Serca}=\frac{F\cdot Vol}{A}\cdot \upsilon_{ER}\cdot \frac{{\left[Ca^{2+}\right]}_{i}^{2}}{{\left[Ca^{2+}\right]}_{i}^{2}+K_{ER}^{2}}. \end{array}$$ (9)

The maximal rate of Ca^2+^ uptake by the SERCA pump is determined by v_ER_. K_ER_ determines the Ca^2+^ affinity of the SERCA pump. In order to relate the current strength to the volume of the intracellular space, the current is multiplied with the volume *Vol*. The current is normalized by the area *A*.

The SERCA current depends on the intracellular Ca^2+^ concentration [Ca^2+^]_i_ and is modeled by a Hill rate expression with an exponent 2. Values of model parameters can be found in Table 3.

*Ca^2+^ leak from the ER*. The Ca^2+^ leak from the endoplasmatic reticulum was taken from [14]:

$$\begin{array}{c} I_{C_{ER}leak}=\frac{F\cdot Vol}{A}\cdot r_{L}\cdot \left({\left[Ca^{2+}\right]}_{ER}-{\left[Ca^{2+}\right]}_{i}\right), \end{array}$$ (10)

where r_L_ is the leak rate.

The leak of Ca^2+^ ions from the endoplasmatic reticulum into the cytosol depends on the difference of the Ca^2+^ concentration in the ER, [Ca^2+^]_ER_, and in the intracellular space [Ca^2+^]_i_. In order to relate the current strength to the volume of the intracellular space, the current is multiplied with the volume *Vol*. The current is normalized by the area *A*. Values of model parameters can be found in Table 3.

#### Transmembrane transporters

*Glutamate transporter*. The transport of glutamate mediated by the glutamate transporter (GluT) is determined by:

$$\begin{array}{c} I_{GluT}=I_{GluT_{max}}\cdot \frac{{\left[K^{+}\right]}_{i}}{{\left[K^{+}\right]}_{i}+K_{GluT_{mK}}}\cdot \frac{{{\left[Na^{+}\right]}_{o}}^{3}}{{{\left[Na^{+}\right]}_{o}}^{3}+{K_{GluT_{mN}}}^{3}}\cdot \frac{g}{g+K_{GluT_{mg}}}, \end{array}$$ (11)

where I_GluT__max_ is the maximal transport current of the glutamate transporter. The half saturation constants of Na^+^, K^+^ and glutamate are given by K_GluT__mN_, K_GluT__mK_ and K_GluT__mg_, respectively. The half saturation constant of K^+^ is not known from experiments. Since the half saturation constant of Na^+^ is close to its intracellular resting concentration, we set the half saturation constant of K^+^ close to its extracellular resting concentration.

The transport of glutamate is coupled to the co-transport of three Na^+^, one Glu^-^ and one H^+^, and the counter-transport of one K^+^ [15, 16]. It results in a net flux of two positive charges per cycle, which is included in the calculation of the membrane potential. The concentrations of H^+^ and Glu^-^ in the different compartments, however, are excluded from the model, because they do not influence any of the other model variables under consideration. Values for the model parameters are listed in Table 5. Additionally, there is a non-stochiometric anion (Cl^-^) current coupled to the glutamate transporter [17]. Inclusion of this current into the equation for the membrane voltage, however, led to minor changes in the simulation results, as long as its maximum conductance was chosen with a physiologically reasonable range (10^-7^ $\frac{nS}{\mu m^{2}}$). It was, therefore, not considered further.

**Table 5:** Model parameters for the currents through the plasma membrane: The glutamate transporter, the Na^+^/K^+^ ATPase, the Na^+^/Ca^2+^ exchanger and the leak currents for Na^+^ and K^+^. The determination of the model parameters I_GluT__max_ and I_NKA__max_ can be found in the Results section **Na^+^ transport by the glutamate transporter**. The definition of the model parameter K_GluT__mK_ can be found in the Model section **Glutamate Transporter**. For the calculation of g_Na__leak_ and g_K__leak_ see Model section **Model parameter values**.

Parameter	Value	Source
**Glutamate Transporter**		
I_GluT__max_	0.68 $\frac{pA}{\mu m^{2}}$	see text
K_GluT__mN_	15 mM	[27]
K_GluT__mK_	5 mM	see text
K_GluT__mg_	34 *μ*M	[27]
**Na^+^/K^+^ ATPase**		
I_NKA__max_	1.52 $\frac{pA}{\mu m^{2}}$	see text
K_NKA__mN_	10 mM	[18]
K_NKA__mK_	1.5 mM	[18]
**Na^+^/Ca^2+^ exchanger**		
I_NCX__max_	0.1 $\frac{pA}{\mu m^{2}}$	see text
K_NCX__mN_	87500 *μ*M	[18]
K_NCX__mC_	1380 *μ*M	[18]
k_sat_	0.1	[18]
*η*	0.35	[18]
**Leak Currents**		
g_Na__leak_	0.0065 $\frac{nS}{\mu m^{2}}$	see text
g_K__leak_	0.0791 $\frac{nS}{\mu m^{2}}$	see text

*Na^+^/K^+^-ATPase*. The transport of Na^+^ and K^+^ against its concentration gradient is performed by the Na^+^/K^+^-ATPase (NKA). We applied the mathematical expression of [18] in a simplified form:

$$\begin{array}{cc} I_{NKA} & =I_{NKA_{max}}\cdot \frac{{\left[Na^{+}\right]}_{i}{}^{1.5}}{{\left[Na^{+}\right]}_{i}{}^{1.5}+K_{NKA_{mN}}^{1.5}}\cdot \frac{{\left[K^{+}\right]}_{o}}{{\left[K^{+}\right]}_{o}+K_{NKA_{mK}}}. \end{array}$$ (12)

Here, I_NKA__max_ defines the maximal pumping activity of the NKA. K_NKA__mN_ and K_NKA__mK_ determine the half saturation constants of Na^+^ and K^+^, respectively.

The Na^+^/K^+^-ATPase (NKA) transports three Na^+^ ions out of the cell and two K^+^ ions into the cell. Its pumping activity depends on the intracellular Na^+^ concentration [Na^+^]_i_ and the extracellular K^+^ concentration [K^+^]_o_ [19]. Values of model parameters can be found in Table 5.

*Na^+^/Ca^2+^ exchanger*. The Na^+^/Ca^2+^ exchanger (NCX) mediates the exchange of three Na^+^ ions with one Ca^2+^ ion. We applied the mathematical description of the NCX of [18]:

$$\begin{array}{c} \begin{array}{cc} I_{NCX} & =I_{NCX_{max}}\cdot \frac{{{\left[Na^{+}\right]}_{o}}^{3}}{{{K_{NCX}}_{mN}}^{3}+{{\left[Na^{+}\right]}_{o}}^{3}}\cdot \frac{{\left[Ca^{2+}\right]}_{o}}{{K_{NCX}}_{mC}+{\left[Ca^{2+}\right]}_{o}}\cdot \\ & \frac{\frac{{{\left[Na^{+}\right]}_{i}}^{3}}{{{\left[Na^{+}\right]}_{o}}^{3}}\cdot exp\left(\eta \cdot \frac{V\cdot F}{R\cdot T}\right)-\frac{{\left[Ca^{2+}\right]}_{i}}{{\left[Ca^{2+}\right]}_{o}}\cdot exp\left(\left(\eta -1\right)\cdot \frac{V\cdot F}{R\cdot T}\right)}{1+k_{sat}\cdot exp\left(\left(\eta -1\right)\cdot \frac{V\cdot F}{R\cdot T}\right)} \end{array} \end{array}$$ (13)

I_NCX__max_ is the maximal pump current of the exchanger. The half saturation constants for Na^+^ and Ca^2+^ are given by K_NCX__mN_ and K_NCX__mC_. The position of the energy barrier *η* controls the voltage dependence. k_sat_ is a saturation factor ensuring saturation at large negative potentials.

The exchanger works either in the forward or in the reverse mode. In the forward mode Ca^2+^ is transported out of the astrocyte and Na^+^ is transported into the astrocyte. The reverse mode works the other way round. A switch into the reverse mode is induced by an increased intracellular Na^+^ concentration [20]. The current strength of the NCX depends on the intra- and extracellular Na^+^ and Ca^2+^ concentrations [Na^+^]_i_, [Na^+^]_o_, [Ca^2+^]_i_ and [Ca^2+^]_o_. Values of model parameters can be found in Table 5.

*Leak currents*. The leak currents of Na^+^ and K^+^ are given by:

$$\begin{array}{c} I_{Na_{leak}}=g_{Na_{leak}}\cdot \left(V-E_{Na}\right) \end{array}$$ (14)

$$\begin{array}{c} I_{K_{leak}}=g_{K_{leak}}\cdot \left(V-E_{K}\right), \end{array}$$ (15)

where g_Na_leak__ and g_K_leak__ are the corresponding conductances of the Na^+^ and K^+^ currents. The Nernst potentials of Na^+^ and K^+^ are E_Na_ and E_K_. Values of model parameters can be found in Table 5.

### Neuronal stimulation of the astrocyte compartment

The release of glutamate from an activated nearby synapse is calculated using the Tsodykis and Makram model [21, 22] in its adapted form published by Wallach and colleagues [7].

$$\begin{array}{ll} r\left(t\right) & =x\left(t\right)\cdot y\left(t\right)\\ \frac{dx}{dt} & =\frac{\left(1-x\left(t\right)\right)}{\tau_{rec}}-x\left(t\right)\cdot y\left(t\right)\cdot s\left(t\right)\\ \frac{dy}{dt} & =-\frac{y\left(t\right)}{\tau_{facil}}+U_{0}\left(1-y\left(t\right)\right)\cdot s\left(t\right)\\ \frac{dg}{dt} & =-\frac{g}{\tau_{clear}}+\rho_{C}G_{T}\cdot r\left(t\right), \end{array}$$

where x and y represent the fraction of resources in the recovered and active states, respectively. During each spike a fraction of active synaptic resources is released into the synaptic cleft, and the time constant *τ*_*rec*_ determines the recovery of these resources. The fraction of active synaptic resources y increases with each spike and the step increase of y is determined by *U*_0_. In the absence of a spike y decays back to a baseline level with time constant *τ*_*facil*_. The product *r*(*t*) corresponds to the ratio of glutamate (g) which is released during a spike of the sequence *s*. The change of the glutamate concentration in the synaptic cleft is determined by the total glutamate content of readily releasable vesicles (*G*_*T*_) and the volume ratio between the synaptic vesicles and the synaptic cleft (*ρ*_*C*_). Glutamate is removed from the synaptic cleft with the time constant *τ*_*clear*_. Values of model parameters can be found in Table 6.

**Table 6:** Parameters for the Tsodyks and Markram model.

Parameter	Value	Source
*τ*_*facil*_	2 *s*^−1^	[7]
*τ*_*rec*_	1 *s*^−1^	[7]
*τ*_*clear*_	60 *s*^−1^	[7]
U_0_	0.25	[7]
*ρ*_C_	6.5 ⋅ 10^−4^	[7]

### Model parameter values

The initial values of [IP_3_]_i_, the fraction h of active IP_3_ receptor channels, and [Ca^2+^]_ER_ and the model parameters g_Na__leak_ and g_K__leak_ were determined as follows. Since the model parameters for the production and degradation of IP_3_ and the intracellular resting concentration of Ca^2+^ were known from literature, the zero of d[IP_3_]_i_/dt revealed the initial concentration of IP_3_. In the same way the initial ratio of activated IP_3_ receptor channels, h, and the initial concentration of the Ca^2+^ concentration in the endoplasmatic reticulum was calculated. In this way a stable resting state was ensured. The model parameter g_Na__leak_ was calculated by setting d[Na^+^]_i_/dt equal to zero and solving the equation for g_Na__leak_. The model parameter g_K__leak_ was calculated the same way by setting d[K^+^]_i_/dt equal to zero.

### Computational methods

All simulations were performed with Python 2.7 using the packages Brian [28], NumPy and Matplotlib. The Brian Simulator used the Euler integration as numerical integration method for the non-linear differential equations with time step dt = 1ms.

## Results

### Influence of ratio_ER_ on the mGluR-driven Ca^2+^ oscillations

**Fig 4:**
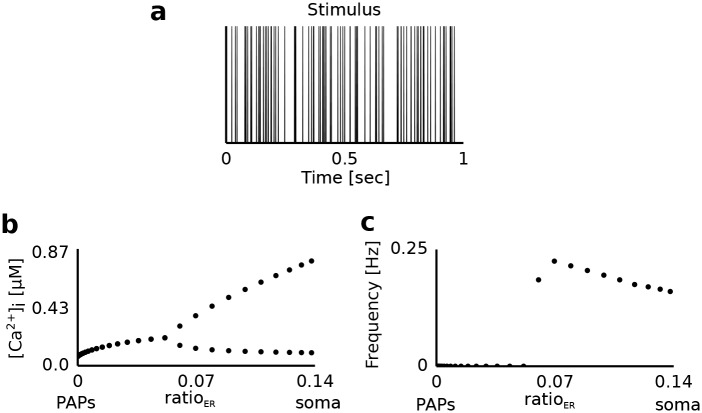
Dynamics of the Ca^2+^ concentration in the intracellular compartment during synaptic activation. **a** Sample stimulus (spikes). The astrocytic compartment was stimulated for 200 seconds with a Poisson spike train of 100 Hz. The corresponding glutamate concentration in the extracellular compartment as a function of time was calculated using the Tsodyks-Markram model. **b** [Ca^2+^]_i_ for different values of the volume ratio (ratio_ER_) between the internal Ca^2+^ store and the intracellular compartments. The upper and lower symbols for ratio_ER_>0.06 denote the average height of peaks and troughs of the emerging Ca^2+^ oscillations (in [*μ*M]). For ratio_ER_≤0.06 no Ca^2+^ oscillations were present and symbols denote the average concentration of Ca^2+^ over the stimulation period. **c** Frequency of Ca^2+^ oscillations as a function of ratio_ER_.

First, we analyzed the generation of mGluR-dependent Ca^2+^ signals along the astrocytic process. For this reason we varied the volume fraction of the internal Ca^2+^ store (ratio_ER_), which changes along the astrocytic process (Fig 3), and studied the amplitude and the frequency of the Ca^2+^ signals (Fig 4). All currents related to the GluT-dependent pathway (*I*_*GluT*_, *I*_*NKA*_, *I*_*NCX*_) were set to zero.

Astrocytic compartments with a high volume fraction of the internal Ca^2+^ store (ratio_ER_>0.06) showed Ca^2+^ oscillations (Fig 4b). These compartments corresponded to astrocytic regions close to the soma. A reduction of ratio_ER_ decreased the amplitude of the Ca^2+^ oscillations. This was caused by the weaker Ca^2+^ influx into the cytoplasm through the smaller surface area of the internal Ca^2+^ store. Astrocytic compartments closer to the synapse (0<ratio_ER_<0.06) did not show Ca^2+^ oscillations, but an increase of the intracellular Ca^2+^ concentration. However, when the astrocytic compartment was devoid of the internal Ca^2+^ store (ratio_ER_ = 0), we observed an unchanged intracellular Ca^2+^ concentration. Different stimulation frequencies led to qualitatively similar behavior (data not shown). In particular, the critical value of ratio_ER_ = 0.06 for the onset of oscillations remained the same.

### Na^+^ transport by the glutamate transporter of the GluT-dependent pathway

The Ca^2+^ entry through the plasma membrane mediated by the Na^+^/Ca^2+^ exchanger is driven by a Na^+^ accumulation in the intracellular space. The glutamate transporter (GluT), the Na^+^/Ca^2+^ exchanger (NCX) and the Na^+^-K^+^-ATPase (NKA) determine the intracellular Na^+^ concentration. For this reason we analyzed the increase of the intracellular Na^+^ concentration as a function of the maximal pump currents of the glutamate transporter (I_GluT__max_), the Na^+^-K^+^-ATPase (I_NKA__max_), and the Na^+^/Ca^2+^ exchanger (I_NCX__max_).

**Fig 5:**
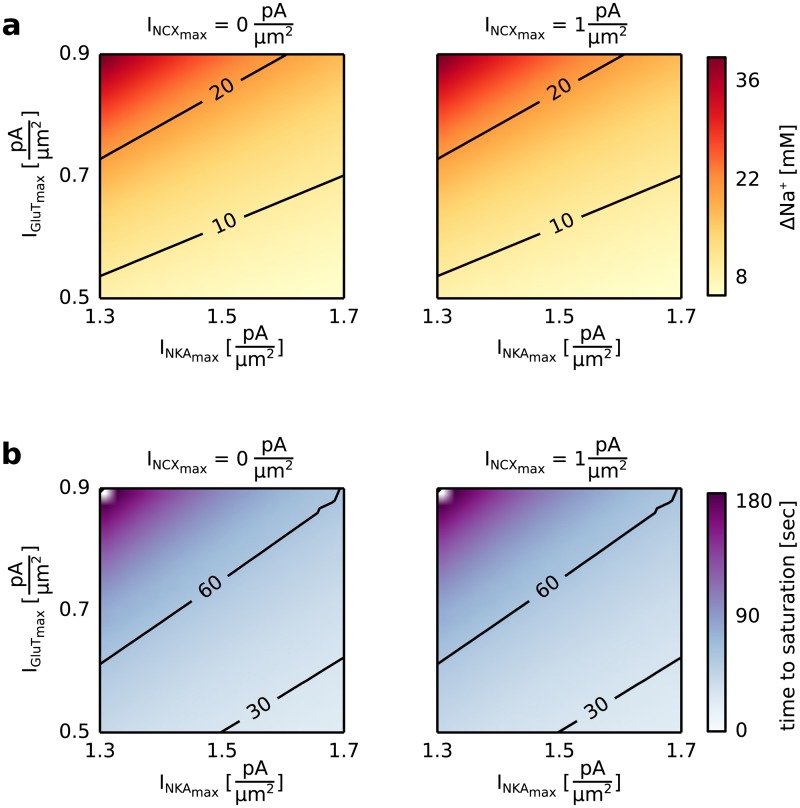
Increase of the Na^+^ concentration in the intracellular compartment, [Na^+^]_i_, during a constant extracellular glutamate concentration for different values of the maximal pump currents of the Na^+^/Ca^2+^ exchanger (I_NCX__max_), the glutamate transporter (I_GluT__max_), the Na^+^/K^+^-ATPase (I_NKA__max_). The astrocytic compartment was stimulated for 200 seconds with a constant extracellular glutamate concentration of 100 *μ*M. The surface volume ratio (SVR) was set equal to 1 *μ*m^-1^, which corresponds to astrocytic compartments close to the soma. **a** [Na^+^]_i_ after 200 seconds with respect to its resting concentration ([Na^+^]_rest_ = 15 mM, Δ Na^+^ = [Na^+^]_End_—[Na^+^]_rest_) for a maximal pump current of the Na^+^/Ca^2+^ exchanger (I_NCX__max_) equal to 0 $\frac{pA}{\mu m^{2}}$ (left) or equal to 1 $\frac{pA}{\mu m^{2}}$ (right) and different values of the maximal pump current of the glutamate transporter (I_GluT__max_) and the Na^+^/K^+^-ATPase (I_NKA__max_). **b** Time to reach saturation for a maximal pump current of the Na^+^/Ca^2+^ exchanger (I_NCX__max_) equal to 0 $\frac{pA}{\mu m^{2}}$ (left) or equal to 1 $\frac{pA}{\mu m^{2}}$ (right) and different values of the maximal pump current of the glutamate transporter (I_GluT__max_) and the Na^+^/K^+^-ATPase (I_NKA__max_). The time to saturation was defined as the time required for the intracellular Na^+^ concentration to remain on a constant concentration.

The maximal pump current of the GluT (I_GluT__max_) and the NKA (I_NKA__max_) had a strong effect on the accumulation of Na^+^ in the astrocyte, while changes of the maximal pump current of the NCX (I_NCX__max_) showed no effect (see Fig 5). The accumulation of Na^+^ in the intracellular space was highest for a high maximal pump current of GluT and a low maximal pump current of NKA (see Fig 5a). While the GluT transported Na^+^ into the astrocyte, the NKA counteracted this effect by pumping Na^+^ out of the astrocyte and led to a saturation of [Na^+^]_i_ at lower concentration levels. The time until saturation was lowest for a low maximal pump current of the GluT and a high maximal pump current of the NKA (see Fig 5b). A low maximal pump current of the GluT resulted in a small Na^+^ accumulation in the intracellular space, which saturated faster for a high Na^+^ transport out of the astrocyte mediated by the NKA.

In experiments the increase of the intracellular Na^+^ concentration in response to external stimulation with glutamate ranges from 10 mM to 20 mM saturating with increasing glutamate concentrations [29] and is performed in under 60 seconds [30]. For the following simulations we chose a parameter combination of the maximal pump currents of the GluT and the NKA which revealed the desired results for the increase of the intracellular Na^+^ concentration and the time to saturation (I_GluT__max_ = 0.68 $\frac{pA}{\mu m^{2}}$ and I_NKA__max_ = 1.52 $\frac{pA}{\mu m^{2}}$.).

### Ca^2+^ transport through the plasma membrane

As a next step, we analyzed how the Ca^2+^ transport through the membrane mediated on the GluT-dependent pathway affects mGluR-dependent Ca^2+^ signals along the astrocytic process. Different regions of the astrocytic process were simulated by changing the volume fraction of the internal Ca^2+^ store (ratio_ER_). We analyzed the influence of the GluT-dependent pathway on the Ca^2+^ signal by changing the maximal pump currents of the Na^+^/Ca^2+^ exchanger (I_NCX__max_) and the glutamate transporter (I_GluT__max_).

**Fig 6:**
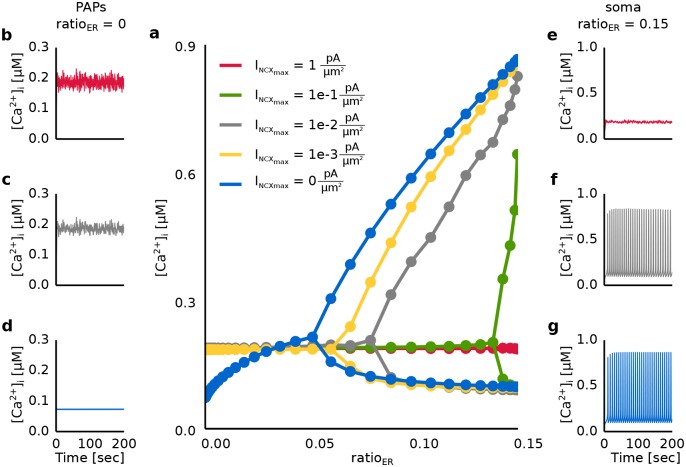
Dynamics of the Ca^2+^ concentration in the intracellular compartment during synaptic activation for different values of the maximal pump current of the Na^+^/Ca^2+^ exchanger (I_NCX__max_). The astrocytic compartment was stimulated for 200 seconds with a Poisson spike train of 100 Hz. The corresponding glutamate concentration in the extracellular compartment as a function of time was calculated using the Tsodyks and Markram model. **a** [Ca^2+^]_i_ as a function of the volume ratio (ratio_ER_) of internal Ca^2+^ stores and the maximal pump current of the Na^+^/Ca^2+^ exchanger (I_NCX__max_). The upper and lower symbols denote the average height of peaks and troughs of the emerging Ca^2+^ oscillations (in [*μ*M]). In case no oscillations were present symbols denote the average concentration of Ca^2+^ over the stimulation period. **b-d** Time course of the Ca^2+^ concentration for ratio_ER_ = 0 and I_NCX__max_ equal to 0 $\frac{pA}{\mu m^{2}}$ (blue), 0.01 $\frac{pA}{\mu m^{2}}$(gray) and 1 $\frac{pA}{\mu m^{2}}$(red). **e-g** Time course of the Ca^2+^ concentration for ratio_ER_ = 0.15 and I_NCX__max_ equal to 0 $\frac{pA}{\mu m^{2}}$ (blue), 0.01 $\frac{pA}{\mu m^{2}}$(gray) and 1 $\frac{pA}{\mu m^{2}}$(red).

First, we analyzed the impact of Ca^2+^ transport through the membrane mediated by the Na^+^/Ca^2+^ exchanger on the intracellular Ca^2+^ signal along the astrocytic process (see Fig 6). During a block of the Ca^2+^ transport through the membrane (I_NCX__max_ = 0 $\frac{pA}{\mu m^{2}}$) Ca^2+^ oscillations were only observed for a high volume fraction of the internal Ca^2+^ store (ratio_ER_>0.06) (see Fig 6a and 6g). An increase of the maximal pump current of the Na^+^/Ca^2+^ exchanger (I_NCX__max_ > 0 $\frac{pA}{\mu m^{2}}$) shifted the critical value of ratio_ER_ for the onset of Ca^2+^ oscillations to higher values (see Fig 6a), culminating in a total suppression of the Ca^2+^ oscillations (see Fig 6e). In astrocytic compartments, which were devoid of the internal Ca^2+^ store (ratio_ER_ = 0), Ca^2+^ was transported into the astrocyte and the intracellular Ca^2+^ concentration increased (see Fig 6b and 6c).

**Fig 7:**
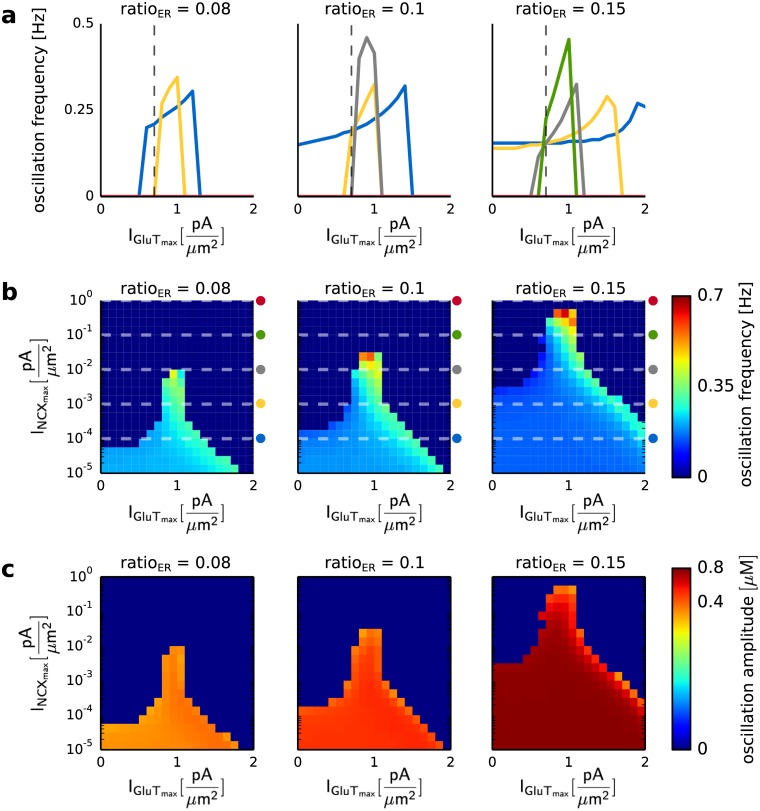
Ca^2+^ oscillation frequency and amplitude for different values of the volume ratio between the internal Ca^2+^ store and the intracellular space (ratio_ER_), as well as the maximal pump currents of the Na^+^/Ca^2+^ exchanger (I_NCX_max__) and the glutamate transporter (I_GluT_max__). The astrocytic compartment was stimulated for 200 seconds with a Poisson spike train of 100 Hz. **a** Ca^2+^ oscillation frequency for three different values of ratio_ER_ (0.08, 0.1 and 0.15), as a function of I_GluT_max__ and I_NCX_max__. The colored lines correspond to I_NCX_max__ equal to 0.0001$\frac{pA}{\mu m^{2}}$ (blue), 0.001$\frac{pA}{\mu m^{2}}$ (yellow), 0.01$\frac{pA}{\mu m^{2}}$ (gray), 0.1$\frac{pA}{\mu m^{2}}$ (green) and 1$\frac{pA}{\mu m^{2}}$ (red). The dashed line corresponds to I_GluT_max__ equal to 0.68$\frac{pA}{\mu m^{2}}$. **b** Ca^2+^ oscillation frequencies for three different values of ratio_ER_ (0.08 0.1 and 0.15), as a function of I_GluT_max__ and I_NCX_max__. The colored symbols denote the values of I_NCX_max__ shown in **a**. **c** Ca^2+^ oscillation amplitudes for four different values of ratio_ER_ (0.05, 0.06, 0.1 and 0.15), and as a function of I_GluT_max__ and I_NCX_max__.

Second, we analyzed the influence of the maximal pump current of the glutamate transporter (I_GluT__max_) on the Ca^2+^ signal (see Fig 7). The impact of I_GluT__max_ on the Ca^2+^ signal mainly depended on the maximal pump current of the Na^+^/Ca^2+^ exchanger (I_NCX__max_) and the volume fraction of the internal Ca^2+^ store (ratio_ER_). In astrocytic compartments close to the soma (ratio_ER_≥0.1) an increase of I_GluT__max_ increased the Ca^2+^ oscillation frequency until it reached a maximal value and decreased again (see Fig 7a and 7b). An increase of I_NCX__max_ shifted the maximal value of the oscillation frequency to lower values of I_GluT__max_ (see Fig 7a). The increase of I_GluT__max_ caused a higher increase of the intracellular Na^+^ concentration. The higher Na^+^ accumulation activated the Na^+^/Ca^2+^ exchanger in the reverse mode and prevented an outflux of Ca^2+^ into the extracellular space. The elevated Ca^2+^ transport into the cell preserved the Ca^2+^ oscillations for high values of I_NCX__max_ and resulted in an increase of the oscillation frequency. The amplitude of the Ca^2+^ oscillations was mainly affected by the volume fraction of internal Ca^2+^ stores and increased with an increase of ratio_ER_ (see Fig 7c). The increase of the volume of both the internal Ca^2+^ store and the intracellular space with ratio_ER_ caused an enhanced Ca^2+^ release from the internal Ca^2+^ store.

The interplay of the mGluR- and GluT-dependent pathways showed the experimentally observed Ca^2+^ fluctuations in astrocytic compartments with a low volume fraction of an internal Ca^2+^ store (ratio_ER_) for a high pumping activity of the NCX (I_NCX__max_ > 0 $\frac{pA}{\mu m^{2}}$). However, a high maximal pump current of the NCX (I_NCX__max_ > 0.01 $\frac{pA}{\mu m^{2}}$) evoked a suppression of the Ca^2+^ oscillations in regions with a high ratio_ER_. Thus, in comparison with experimental data the simulation data suggested a low maximal pump current of the NCX for regions with a high ratio_ER_ and a high maximal pump current of the NCX in regions with a small ratio_ER_. Moreover, an increase of I_GluT__max_ allowed Ca^2+^ oscillations for high values of I_NCX__max_ (I_NCX__max_ ≥ 1 $\frac{pA}{\mu m^{2}}$). Thus, the distribution of GluTs and NCXs determines Ca^2+^ signal along the astrocytic process. The reason for the suppression of the Ca^2+^ oscillations for high ratio_ER_ was investigated in a later results section.

### Impact of the GluT activity on the Ca^2+^ response under synaptic stimulation

**Fig 8:**
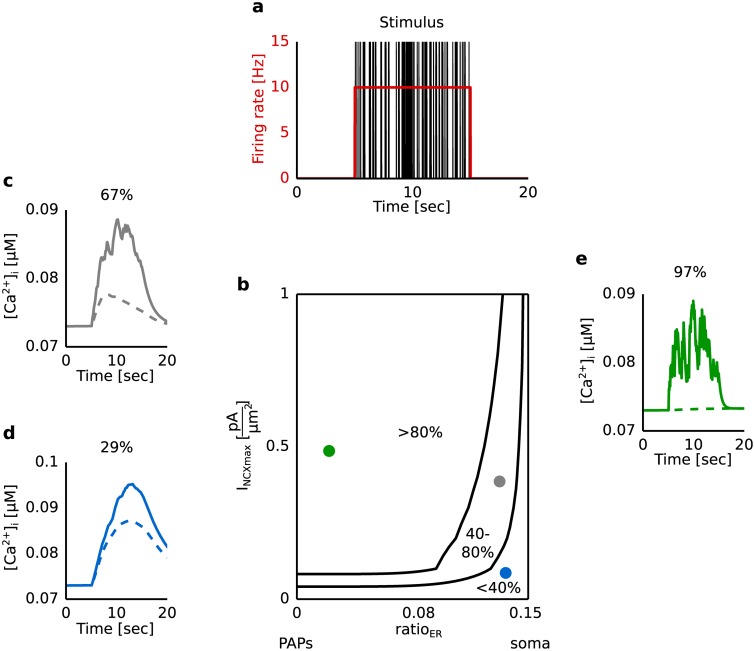
Dynamics of the Ca^2+^ concentration in the intracellular compartment under synaptic stimulation for a blocked glutamate transporter (GluT) in comparison to the control condition. **a** The astrocytic compartment was stimulated for 10 seconds with a Poisson spike train of 10 Hz. The corresponding glutamate concentration in the extracellular compartment as a function of time was calculated using the Tsodyks and Markram model. **b** Reduction of the Ca^2+^ response under block of the GluT as a function of the maximal pump current of the Na^+^/Ca^2+^ exchanger (I_NCX__max_) and the volume ratio (ratio_ER_) between the internal Ca^2+^ store and the intracellular compartment. The reduction was quantified by the difference of the average Ca^2+^ concentration under control condition and block normalized by the difference between the Ca^2+^ concentration in the control condition and the Ca^2+^ concentration without stimulation. Solid lines separate the parameter space concerning the reduction: larger than 80%, between 40% and 80% and under 40%. **c-e** Ca^2+^ response as a function of time for different values of I_NCX__max_ and ratio_ER_ correspond to a reduction of the Ca^2+^ signal of 29%, 67% and 97%. Solid and dashed lines correspond to control condition and block. The block was simulated by setting I_GluT__max_ equal to 0 $\frac{pA}{\mu m^{2}}$.

Experiments have shown that a block of the glutamate transporter (GluT) leads to a clear attenuation of the Ca^2+^ signal [8]. For that reason we examined the impact of the GluT-driven Ca^2+^ signal on the overall Ca^2+^ response to synaptic stimulation. Fig 8 shows the dynamics of the Ca^2+^ signal as a function of the volume ratio between the internal Ca^2+^ store and the intracellular space (ratio_ER_) with (’control condition’) and without (’block’) a contribution of the GluT.

We observed a high impact of the GluT-driven Ca^2+^ signal for a high pumping activity of the Na^+^/Ca^2+^ exchanger (I_NCX__max_ > 0.1 $\frac{pA}{\mu m^{2}}$) and a small volume ratio between the internal Ca^2+^ store and the intracellular space (ratio_ER_<0.1) (see Fig 8b and 8e). With a decrease of I_NCX__max_ and an increase of ratio_ER_ the impact of the GluT-driven Ca^2+^ signal decreased (see Fig 8b, 8c and 8d). In astrocytic compartments with a low volume fraction of the internal Ca^2+^ store the Ca^2+^ signal mainly arose by the Ca^2+^ transported through the membrane (see Fig 4). A block of the glutamate transporter prevented a Na^+^ accumulation in the intracellular space (see S1 Fig). The Na^+^/Ca^2+^ exchanger remained in the forward mode and transported Ca^2+^ out of the astrocyte. Thus, during a block of the glutamate transporter no Ca^2+^ was transported into the astrocyte via the Na^+^/Ca^2+^ exchanger and a clear attenuation of the Ca^2+^ signal was observed in regions with a small ratio_ER_. With an increase of the volume fraction of the internal Ca^2+^ store more Ca^2+^ was released from the internal Ca^2+^ store and led to a lower impact of the glutamate transporter on the overall Ca^2+^ signal. The extracellular glutamate concentration and the Ca^2+^ entry through the membrane affected the IP_3_ production as well as the IP_3_- and Ca^2+^-dependent Ca^2+^ release from internal Ca^2+^ stores. An increase of ratio_ER_ was accompanied with an increase of the IP_3_- and Ca^2+^-dependent Ca^2+^ release from internal Ca^2+^ stores and thus with an increase of the impact of the mGluR-dependent mechanism. For that reason, Ca^2+^ signals mainly evoked by the GluT-dependent mechanism were observed in regions with a small ratio_ER_.

### Interaction of the mGluR-dependent and GluT-dependent pathway

**Fig 9:**
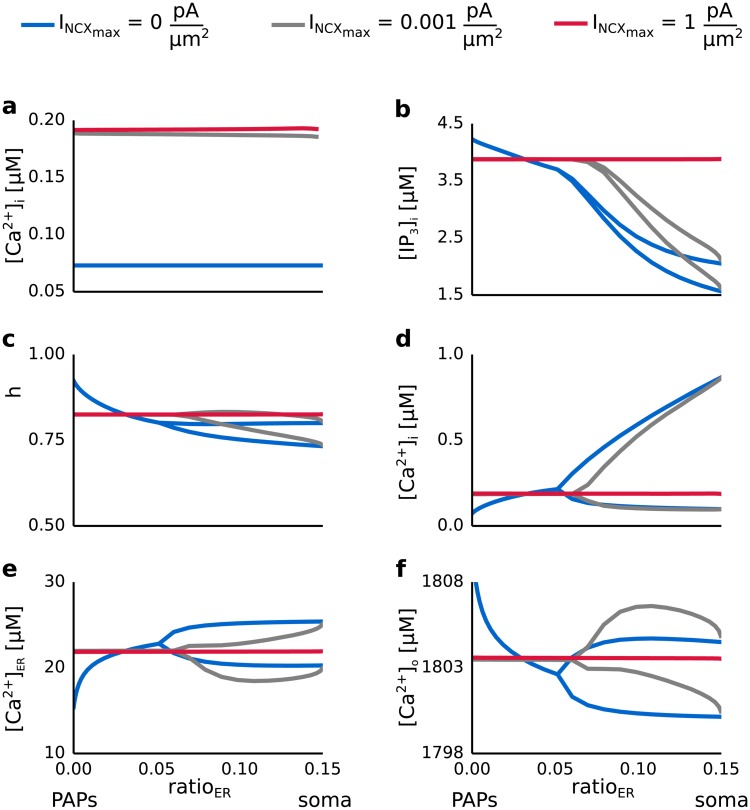
Ca^2+^ concentrations in the intracellular space, the internal Ca^2+^ store and the extracellular space, the IP_3_ concentration in the intracellular space and the ratio h of active IP_3_ receptor channels as a function of the maximal pump current of the Na^+^/Ca^2+^ exchanger (I_NCX__max_) and the volume ratio between the internal Ca^2+^ store and the intracellular space (ratio_ER_). The astrocytic compartment was stimulated for 200 seconds with a Poisson spike train of 100 Hz. The corresponding glutamate concentration in the extracellular compartment as a function of time was calculated using the Tsodyks and Markram model. Blue, gray and red lines denote the dynamics of [IP_3_]_i_, h [Ca^2+^]_i_, [Ca^2+^]_ER_, [Ca^2+^]_o_ for values of I_NCX__max_ equal to 0 $\frac{pA}{\mu m^{2}}$, 0.001 $\frac{pA}{\mu m^{2}}$ and 1 $\frac{pA}{\mu m^{2}}$. **a** Analysis of the GluT-dependent pathway in isolation. [Ca^2+^]_i_ is shown as a function of ratio_ER_ and for different values of I_NCX__max_. **b-f** Analysis of both the mGluR- and the GluT-dependent pathway. [IP_3_]_i_, h, [Ca^2+^]_i_, [Ca^2+^]_ER_ and [Ca^2+^]_o_ are shown as a function of ratio_ER_ and for different values of I_NCX__max_.

In order to study the mechanisms underlying the interaction of the mGluR- and GluT-dependent pathways we analyzed the Ca^2+^ concentration in the three spaces as well as the concentration of IP_3_ in the intracellular space and the fraction h of open IP_3_ channels for different values of the maximal pump current of the Na^+^/Ca^2+^ exchanger (I_NCX__max_) and the volume ratio of internal Ca^2+^ stores (ratio_ER_). Fig 9 summarizes the results. Oscillations of the Ca^2+^ concentration in the intracellular compartment (see Fig 9b) were reflected in all of the other dynamical variables (see Fig 9c–9f). When the GluT-dependent pathway was studied in isolation and Ca^2+^ release from internal Ca^2+^ stores was neglected (see Fig 9a) a finite current through the Na^+^/Ca^2+^ exchanger led to an increase of [Ca^2+^]_i_ when compared with the concentration without external stimulation. The stationary value of [Ca^2+^]_i_ was independent of the maximal pump currents.

When both the GluT- and mGluR-dependent pathway were considered (see Fig 9b–9f) a high I_NCX__max_ (I_NCX_max__ > 0.001$\frac{pA}{\mu m^{2}}$) caused an increase of the concentration of IP_3_ and the fraction h of open IP_3_ receptor channels. This caused Ca^2+^ flux out of the internal Ca^2+^ store leading to a decrease of [Ca^2+^]_ER_ compared to the resting concentration. The concentration of Ca^2+^ in the intracellular space, however, increased by 0.1 *μM* while [Ca^2+^]_o_ increased by 3 *μM* compared to its resting concentration. For high values of the maximal pump current of the Na^+^/Ca^2+^ exchanger the Ca^2+^ transport into the endoplasmatic reticulum mediated by the SERCA pump was overcompensated by the highly strong outflux of Ca^2+^ via the Na^+^/Ca^2+^ exchanger (see **S??**). Thus, Ca^2+^ accumulated in the extracellular space, which prevented the generation of Ca^2+^ oscillations.

## Discussion

Our computational study addresses the generation of Ca^2+^ signals in different astrocytic compartments along the astrocytic process. We considered two different pathways for the generation of Ca^2+^ signals: the metabotropic glutamate receptor (mGluR)- and glutamate transporter (GluT)-dependent pathway. We analyzed both pathways in consideration of the volume ratio between the internal Ca^2+^ store and the intracellular space. The volume ratio between the internal Ca^2+^ store and the intracellular space changes from the soma towards the synapse. Whereas astrocytic compartments at the soma have a high volume ratio between the internal Ca^2+^ store and the intracellular space, in astrocytic compartments close to the synapse there is a low volume ratio. There are five main findings of the study.

First, while considering the mGluR-dependent pathway in isolation Ca^2+^ oscillations have only been observed in astrocytic compartments with a high volume ratio between the internal Ca^2+^ store and the intracellular space. Second, a high maximal pump current of the Na^+^/Ca^2+^ exchanger suppressed Ca^2+^ oscillations in regions with a high volume ratio between the internal Ca^2+^ store and the intracellular space. Third, the suppression of Ca^2+^ oscillations for a high maximal pump current of the Na^+^/Ca^2+^ exchanger in astrocytic compartments with a high volume ratio between the internal Ca^2+^ store and the intracellular space was due to an overcompensation of the Ca^2+^ influx from the internal Ca^2+^ store by the outflux of Ca^2+^ into the extracellular space via the Na^+^/Ca^2+^ exchanger. Fourth, a high impact of the GluT-dependent mechanism on the generation of Ca^2+^ signals was observed for a high maximal pump current of the Na^+^/Ca^2+^ exchanger in regions with a low volume ratio between the internal Ca^2+^ store and the intracellular space. Fifth, the GluT-dependent mechanism accounted for Ca^2+^ fluctuations in astrocytic compartments which were devoid of internal Ca^2+^ stores.

In their study Srinivasan and colleagues also addressed the question which mechanism could account for Ca^2+^ fluctuations in astrocytic compartments close to the synapse. They discovered that a significant proportion of Ca^2+^ signals in astrocytic compartments close to the synapse is because of transmembrane Ca^2+^ fluxes. In our model we also considered Ca^2+^ transport from the extracellular space into the intracellular space of the astrocyte through the GluT-dependent pathway. We found that the GluT-dependent Ca^2+^ transport into the astrocyte could account for mGluR-independent Ca^2+^ fluctuations in astrocytic compartments with a low volume ratio between the internal Ca^2+^ store and the intracellular space.

However, while analyzing both the mGluR- and GluT-dependent pathway a high maximal pump current of the Na^+^/Ca^2+^ exchanger suppressed Ca^2+^ oscillations in astrocytic compartments with a high volume ratio between the internal Ca^2+^ store and the intracellular space. Moreover, the contribution of the GluT on the generation of Ca^2+^ signals was highest for a large maximal pump current of the Na^+^/Ca^2+^ exchanger in astrocytic compartments with a low volume ratio between the internal Ca^2+^ store and the intracellular space. These simulation results suggested a change of the pumping activity of the Na^+^/Ca^2+^ exchanger along the astrocytic process. A low maximal pump current in astrocytic compartments at the soma prevented the suppression of Ca^2+^ oscillations. A high maximal pump current in astrocytic compartments close to the synapse allowed a high contribution of the GluT-dependent pathway on the generation of Ca^2+^ signals. Based on the strength of the maximal pump current the channel density of the Na^+^/Ca^2+^ exchanger can be concluded. The higher the maximal pump current is, the more ions are transported through the membrane. The same holds true for the channel density. The higher the channel density is, the more ions are transported through that channel. Experimental results confirm a concentration and colocalization of Na^+^/Ca^2+^ exchangers, Na^+^/K^+^-ATPases and GluTs in perisynaptic astrocytic processes [31, 32].

Ca^2+^ transport through the plasma membrane (e.g. via the Na^+^/Ca^2+^ exchanger) [23, 33, 34] as well as by the Ca^2+^ diffusion within a single astrocyte [35, 36] or between astrocytes [37] changes the intracellular Ca^2+^ concentration. Fluctuations of the intracellular Ca^2+^ concentration affect both the Ca^2+^ entry mediated by the Na^+^/Ca^2+^ exchanger when operating in the reverse mode [23] and the Ca^2+^ release probability of the endoplasmatic reticulum [38]. The current model neglects Ca^2+^ diffusion within the astrocyte and describes the Ca^2+^ dynamics in a single compartment. Thus, an extension of the current point-model to a multi-compartment model will most probably reveal deviating results for parameters such as the maximal pump current of the Na^+^/Ca^2+^ exchanger. Moreover, the volume determines the number of Ca^2+^ ions within an astrocytic compartment and consequently the concentration change. Thus, diffusion of Ca^2+^ in astrocytic compartments with a low volume, such as in the perisynaptic astrocytic processes, leads to a bigger concentration change as in compartments with a larger volume.

The above named findings allow to make a prediction about the functional role of astrocytes in neural networks. Astrocytic compartments, which have a high volume ratio of internal Ca^2+^ stores and are capable of IP_3_-dependent Ca^2+^ release, are not located directly at the synapse. Moreover, the high surface volume ratio of the perisynaptic astrocytic processes and a slow diffusion exchange in such thin processes favors a localized Na^+^ accumulation and promotes Ca^2+^ intrusion mediated by the NCX [39]. This may indicate that store-dependent Ca^2+^ signals in astrocytes act as integrators of local network activity, but not as detectors of individual synaptic events [12]. GluT-dependent Ca^2+^ signals in perisynaptic astrocytic processes are evoked in response to individual synaptic events. Depending on the synaptic activity Ca^2+^ is transported into the astrocyte by the Na^+^/Ca^2+^ exchanger and diffuses within the astrocyte network. Once this Ca^2+^ wave reaches astrocytic compartments which are capable of store dependent Ca^2+^ signals an integration of the local network activity, the intracellular Ca^2+^ signal and the glutamate-dependent IP_3_ production, takes place.

Our model describes the generation of Ca^2+^ signals in a single astrocyte compartment with respect to its morphology. However, it is of special interest how activity of single synapses and neural networks is integrated by astrocytes. It was proposed that perisynaptic astrocytic processes serve as detectors for single synaptic events, whereas astrocytic processes which contain Ca^2+^ stores act as integrators of neural network activity [12]. A multi-compartment model would contribute to the analysis of the integration of neural activity performed by astrocytes. This would allow the study of Ca^2+^ waves within a single astrocyte and in astrocyte networks as well as their impact on the surrounding extracellular space.

## Supporting information

S1 FigIncrease of the intracellular Na^+^ concentration during synaptic stimulation.Dynamics of the Na^+^ concentration in the intracellular compartment under synaptic stimulation for a blocked glutamate transporter in comparison to the control condition. The astrocytic compartment was stimulated for 10 seconds with a Poisson spike train of 10 Hz (see Fig 8). **a** Time course of the glutamate concentration in the extracellular compartment calculated with the Tsodyks and Markram model. **b-d** Time course of the intracellular Na^+^ concentration for three different parameter combinations of the volume ratio between the internal Ca^2+^ store and the intracellular space (ratio_ER_) and the maximal pump current of the Na^+^/Ca^2+^ exchanger (I_NCX_max__). The intracellular Na+ concentration is shown for the same parameter combinations of I_NCX_max__ and ratio_ER_ as Ca^2+^ in Fig 8c, 8d and 8e (**b**: ratio_ER_ = 0.14 and I_NCX_max__ = 0.1 $\frac{pA}{\mu m^{2}}$, **c**: ratio_ER_ = 0.12 and I_NCX_max__ = 0.4 $\frac{pA}{\mu m^{2}}$, **d**: ratio_ER_ = 0.03 and I_NCX_max__ = 0.5 $\frac{pA}{\mu m^{2}}$). Solid and dashed lines corresponds to the control condition and block, respectively. The intracellular Na^+^ concentration was not affected by different values of ratio_ER_ and I_NCX_max__. During a block of the glutamate transporter (dashed lines) the Na^+^ concentration remained on its resting concentration.(EPS)

S2 Fig Impact of the stimulation frequency on the Ca^2+^ oscillation frequency. Ca^2+^ oscillation frequency as a function of the maximal pump current of the Na^+^/Ca^2+^ exchanger (I_NCX_max__) and the stimulation frequency, as well as for different values of the volume fraction of the internal Ca^2+^ store (ratio_ER_). The astrocytic compartment was stimulated for 200 seconds with a Poisson spike train of 5-100 Hz. The corresponding glutamate concentration was calculated using the Tsodyks Markram model. The parameter space for which Ca^2+^ oscillations were observed increased with an increase of ratio_ER_. An onset of the Ca^2+^ oscillations was observed for stimulation frequencies greater than 5 Hz. The oscillation frequency, however, decreased for an increase of the volume fraction of internal Ca^2+^ stores. Thus, a larger volume of the internal Ca^2+^ store and the intracellular space favored the generation of Ca^2+^ oscillations and a longer Ca^2+^ oscillation period.
(EPS)

S3 Fig Impact of the stimulation frequency on the current strength of the Na^+^/Ca^2+^ exchanger. Current strength of the Na^+^/Ca^2+^ exchanger (I_NCX_) as a function of the maximal pump current of the Na^+^/Ca^2+^ exchanger (I_NCX_max__) and the stimulation frequency, as well as for different values of the volume fraction of the internal Ca^2+^ store (ratio_ER_). The astrocytic compartment was stimulated for 200 seconds with a Poisson spike train of 5-100 Hz. The corresponding glutamate concentration was calculated using the Tsodyks Markram model. The white area corresponds to parameter combinations which evoked Ca^2+^ oscillations. An increase of the volume fraction of the internal Ca^2+^ store led to a decrease of I_NCX_, which corresponded to a larger outflux of Ca^2+^ out of the astrocyte.
(EPS)

S4 Fig Response of the model to a single action potential. Time course of the intracellular Ca^2+^ concentration, the current strengths of the Na^+^/Ca^2+^ exchanger and the IP_3_-receptor current for different values of the maximal pump current of the Na^+^/Ca^2+^ exchanger (I_NCX_max__) and the volume fraction of the internal Ca^2+^ store (ratio_ER_). **a** The astrocytic compartment was stimulated with a single action potential. The gray line corresponds to the time point of the action potential. The corresponding glutamate concentration was calculated using the Tsodyks Markram model. **b-d** Time courses of the intracellular Ca^2+^ concentration [Ca^2+^]_i_, the current strengths of the Na^+^/Ca^2+^ (I_NCX_) and the IP_3_-receptor current (I_IP_3_R_) for different values of I_NCX_max__ and ratio_ER_. After the application of a single action potential the Ca^2+^ concentration returned fastest to the resting concentration when the astrocytic compartment was devoid of the internal Ca^2+^ store (ratio_ER_ = 0) (see **b**). This process was slowed down by the Ca^2+^ transport mechanisms at the internal Ca^2+^ store (ratio_ER_ = 0.06 and 0.15). In general, the current strength of the Na^+^/Ca^2+^ exchanger reached the steady state much faster than the current strength of the IP_3_-receptor current (see **c, d**). With an increase of the volume fraction of the internal Ca^2+^ store also the impact of the Ca^2+^ transport mechanisms at the endoplasmatic reticulum on the intracellular Ca^2+^ concentration increased. Thus, for larger values of ratio_ER_ it took longer for Ca^2+^ to return to its resting concentration.
(EPS)
