# Correction: Spatial separation of two different pathways accounting for the generation of calcium signals in astrocytes

**DOI:** 10.1371/journal.pcbi.1005514

**Published:** 2017-04-27

**Authors:** The PLOS Computational Biology Staff

There is an error in Equation 4. It should read:

$${\left[{Ca}^{2+}\right]}_{o}-{\left[{Ca}^{2+}\right]}_{o_{rest}}={\left[{Ca}^{2+}\right]}_{i_{rest}}-{\left[{Ca}^{2+}\right]}_{i}+{\left[{Ca}^{2+}\right]}_{{ER}_{rest}}-{\left[{Ca}^{2+}\right]}_{ER}$$
